# Efficacy and Safety of Nanosomal Docetaxel Lipid Suspension-Based Chemotherapy in Sarcoma: A Multicenter, Retrospective Study

**DOI:** 10.1155/2019/3158590

**Published:** 2019-11-15

**Authors:** Prasad Narayanan, Palanki Satya Dattatreya, Rammohan Prasanna, Sundaram Subramanian, Kunal Jain, Nirni Sharanabasappa Somanath, Nisarg Joshi, Deepak Bunger, Mujtaba Ali Khan, Alok Chaturvedi, Imran Ahmad

**Affiliations:** ^1^Cytecare Cancer Hospitals, Yelahanka, Bengaluru, Karnataka 560064, India; ^2^Omega Hospitals, Hyderabad, Telangana 500034, India; ^3^Department of Medical Oncology, CBCC-GVN Cancer Center, Tiruchirapalli, Tamil Nadu 620005, India; ^4^VS Hospital, Madras Cancer Institute, Advanced Cancer Care, Chennai, Tamil Nadu 600031, India; ^5^American Oncology Institute, Dayanand Medical College & Hospital, Ludhiana, Punjab 141001, India; ^6^Medical Affairs and Clinical Development, Intas Pharmaceuticals Ltd., Sola, Ahmedabad, Gujarat 380054, India; ^7^Jina Pharmaceuticals Inc., Libertyville, Green Oaks, Illinois 60048, USA

## Abstract

**Objective:**

To evaluate the efficacy and safety of nanosomal docetaxel lipid suspension (NDLS, DoceAqualip) based chemotherapy in patients with sarcoma.

**Methods:**

In this retrospective, multicenter (6 centers), observational study, we analyzed the medical charts of adult patients of either sex, who were treated with NDLS (75 mg/m^2^ in 3-weekly cycles) based chemotherapy for the treatment of sarcoma. The efficacy outcomes were overall response rate (ORR: complete response (CR) + partial response (PR)) and disease control rate (DCR: CR + PR + stable disease (SD)) in patients who received NDLS-based chemotherapy in neoadjuvant and metastatic settings. Overall survival (OS) and safety were evaluated for all settings.

**Results:**

Of 11 patients (neoadjuvant: 1, adjuvant: 3, and metastatic: 7) in this study, majority had leiomyosarcoma (63.6%, 7/11) followed by extraskeletal myxoid chondrosarcoma (EMC), high grade pleomorphic sarcoma of mandible, malignant fibrous histiocytoma of right thigh, and osteosarcoma of femur (9.1% each, 1/11 each). NDLS plus gemcitabine combination was used in 10 patients (90.9%), and NDLS plus cyclophosphamide was used in one patient with EMC (9.1%). Efficacy evaluation was performed for 7 patients (neoadjuvant: 1/1; metastatic: 6/7). Complete response was reported in one patient (soft tissue sarcoma of mandible) treated in neoadjuvant setting. In metastatic setting, ORR was 50% and DCR was 66.7% (CR: 16.7% (1/6), PR: 33.3% (2/6), SD: 16.7% (1/6)). At a median follow-up of 6.5 months (range: 0.06–20.2 months), median OS was not reached in neoadjuvant and adjuvant settings, but it was 15.8 months in metastatic setting. At least 1 AE was reported in 7 (63.6%) patients. Neutropenia, thrombocytopenia, lymphopenia, and anemia were the hematological AEs, whereas nausea, vomiting, and diarrhea were the most common nonhematological AEs. NDLS treatment was well tolerated without any new safety concerns.

**Conclusion:**

Nanosomal docetaxel lipid suspension-based chemotherapy was efficacious and well tolerated in the treatment of sarcoma. Further prospective trials are needed to confirm the data.

## 1. Introduction

Sarcomas are a rare heterogeneous group of solid tumors and are broadly classified as soft tissue sarcomas (STS) and bone sarcomas [1, 2]. The incidence of sarcomas is 2–4/100,000 people [2]. It is common in children accounting for ∼15% of all cancers, whereas it accounts for ∼1% of all cancer cases in adults [3]. The incidence of STS was ∼10% as per Indian reports [4]. The most common sarcoma type is STS, and the most common sites of STS are extremities (lower limb > upper limb); thigh is the commonest site [2]. With >50 subtypes available, the most common STSs are pleomorphic sarcoma, gastrointestinal stromal tumors (GIST), leiomyosarcoma, synovial sarcoma, liposarcoma, and malignant peripheral nerve sheath tumors [3]. Osteosarcomas are the most common bone sarcomas followed by Ewing's sarcomas, and these can present in all bones [5].

Multimodality treatment approach, including surgery, radiation therapy, and chemotherapy, is recommended for sarcomas [6]. Among many regimens used in clinical practice, docetaxel alone [7] or in combination with gemcitabine [8, 9] is recommended by several guidelines [10, 11]. Docetaxel has shown activity in the treatment of sarcomas, but toxicity issues such as hypersensitivity reactions, fluid retention, sensory neurotoxicity, and anaphylactoid reactions observed in these patients [7] are known to be associated with the carrier polysorbate 80 in the conventional docetaxel formulation. A solvent-free lipid-based formulation “nanosomal docetaxel lipid suspension (NDLS, DoceAqualip)” was developed [12], which has shown effectiveness and tolerability in the treatment of several cancers including sarcoma [13]. We report here a multicenter, retrospective experience in real-life practice evaluating the effectiveness and tolerability of NDLS in the treatment of sarcomas.

## 2. Methods

### 2.1. Study Design, Patient Selection, and Endpoints

In this multicenter (6 centers), retrospective, observational study, we analyzed the medical charts of sarcoma patients who received NDLS-based chemotherapy as part of their routine clinical care between February 2016 and March 2019. The study endpoints included overall response rate (ORR: proportion of patients achieving complete response (CR) and partial response (PR)) and disease control rate (DCR: proportion of patients achieving CR + PR + stable disease (SD)) for patients treated in neoadjuvant and metastatic settings, whereas overall survival (time from treatment to death due to any cause) was evaluated for patients treated in all settings. Treatment response was evaluated using Response Evaluation Criteria in Solid Tumors (RECIST) 1.1 [14]. The National Cancer Institute (NCI) Common Terminology Criteria for Adverse Events (CTCAE) Criteria version 5 [15] were used to grade (where available) the incidence of adverse events (AEs) recorded from the treatment charts. Data on death and discontinuations were recorded from patients' medical charts. The study was conducted after due approval from ethics committee and in compliance with the protocol.

### 2.2. Statistical Considerations

Demographic and baseline characteristics were summarized using descriptive statistics. Categorical variables were summarized with frequency and percentage. Continuous variables were summarized with count, mean, standard deviation, median, minimum, and maximum. Response rate was presented as frequency and percentage of patients. *χ*^2^ test was used to compare the distribution of patients in each category. Survival analysis was performed to measure lifetime or the length of time until the occurrence of an event (death in case of overall survival). Survival data were analyzed using a nonparametric procedure which performed PROC LIFETEST of SAS (version 9.4) to measure the duration of time until a specified event occurs. OS was calculated and analyzed using Kaplan–Meier method and log-rank test to estimate the survival function from lifetime data after treatment. The AEs were summarized as frequencies and percentages by type of reactions.

## 3. Results

### 3.1. Patient Disposition and Demographics

Eleven patients with sarcoma, who were treated with NDLS-based chemotherapy, were retrospectively analyzed. Majority of the patients had leiomyosarcoma (63.6%, 7/11). Extraskeletal myxoid chondrosarcoma (EMC), high grade pleomorphic sarcoma of mandible, malignant fibrous histiocytoma of the right thigh, and high grade osteosarcoma of femur were diagnosed in 1 patient each (9.1% each). The baseline characteristics of patients are summarized in Table 1.

NDLS was used as 1 hour infusion in 3-weekly cycles at a dose of 75 mg/m^2^. NDLS was used as a second-line therapy in majority (8, 72.7%) of the patients; 2 patients (18.2%) received as first-line therapy, and one patient (9.1%) as third-line therapy. Most (9, 81.8%) of the patients were administered premedications; dexamethasone premedication was administered to 54.5% patients. All the patients received G-CSF/Peg-GCSF as primary prophylaxis. NDLS was used in combination with gemcitabine (dose range: 600–1100 mg/m^2^) in 90.9% patients and cyclophosphamide (dose: 600 mg/m^2^) in 9.1% patients.  Table 2 presents the patient details with chemotherapy regimens used and efficacy evaluation.

### 3.2. Efficacy

**Figure 1:**
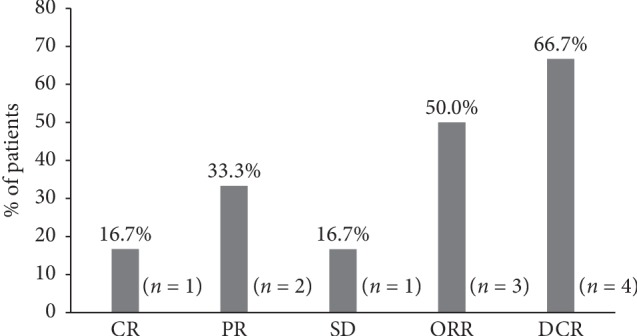
Efficacy of NDLS-based chemotherapy for the treatment of sarcoma in metastatic setting (*n* = 6). CR: complete response; DCR: disease control rate; NDLS: nanosomal docetaxel lipid suspension; ORR: overall response rate; PR: partial response. Disease progression was reported in 2 patients.

Patients who received NDLS-based regimen as adjuvant chemotherapy (*n* = 3) were considered for safety and overall survival analysis. Of 8 patients in neoadjuvant and metastatic settings, efficacy evaluation was available for 7 patients (neoadjuvant: 1 and metastatic: 6). One patient treated in neoadjuvant setting showed complete response (ORR and DCR: 100%). In the metastatic setting, NDLS-based chemotherapy resulted in an ORR of 50% (CR: 16.7% (1/6), PR: 33.3% (2/6)) and DCR of 66.7% (CR: 16.7% (1/6), PR: 33.3% (2/6), SD: 16.7% (1/6)) (Figure 1). Disease progression was reported in 2 patients treated in the metastatic setting.

### 3.3. Overall Survival

**Figure 2:**
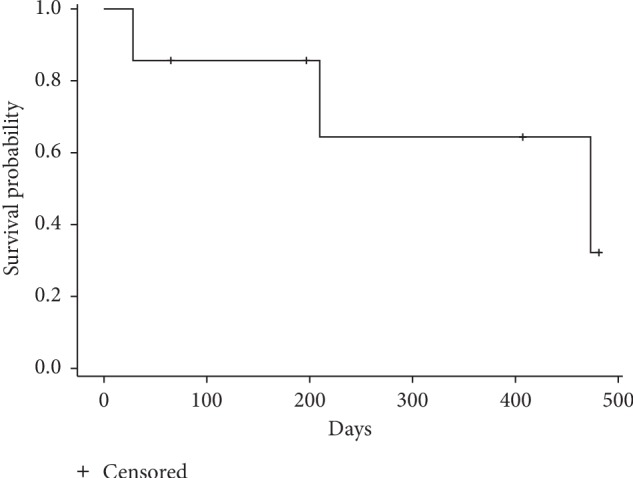
Kaplan–Meier estimates of overall survival in sarcoma in metastatic (*n* = 7) setting.

Overall, patient survival data were collected from the administration of the first dose of NDLS-based therapy till the last follow-up date for alive patients and date of death for patients who died. At a median follow-up of 6.5 months (range: 0.06–20.2 months), there were 3 (18.18%) deaths (metastatic setting: 3 patients), the median OS was not reached in neoadjuvant and adjuvant settings, but it was 15.8 months in metastatic setting (Figure 2).

### 3.4. Safety

At least 1 AE was reported in 7 (63.6%) patients. Grade 1 AEs were reported in 45.5% (5/11) patients, grade 2 in 36.4% (4/11) patients; grade 3/4 AEs were not reported. Neutropenia, thrombocytopenia, lymphopenia, and anemia were the hematological AEs, whereas nausea, vomiting, and diarrhea were the most common nonhematological AEs (Table 3).

## 4. Discussion

The current multicenter, retrospective study demonstrated the effectiveness and tolerability of solvent-free NDLS formulation in patients with sarcoma. NDLS (75 mg/m^2^) as a part of different chemotherapy regimens (NDLS plus gemcitabine/cyclophosphamide) demonstrated complete response in 1 patient treated in neoadjuvant setting and showed an ORR of 50% and DCR of 66.7%, respectively, for patients treated in metastatic setting. A median OS of 15.8 months was reported in the metastatic setting, whereas median OS was not reached in neoadjuvant and adjuvant settings.

NDLS, a lipid-based formulation of docetaxel, was developed with an intent to avoid the toxicity issues related to the carriers (polysorbate 80 and ethanol) used in the conventional formulation. NDLS was developed based on the patented (worldwide (WO2008127358), Europe (2076244), Japan (5917789), and Canada (CA2666322)) “NanoAqualip” technology [16] with generally recognized as safe (GRAS) lipids by the US Food and Drug Administration. The resultant nanosomal particles (<100 nm) [16] may allow the docetaxel infiltration and entrapment in the weakened tumor vasculature and necrotic tumor tissue collagen material, thus causing increased retention (enhanced permeability retention (EPR) effect), leading to a greater systemic availability of docetaxel [16, 17], and ultimately improved outcome [12], which may have potential in the treatment of difficult to treat cancers such as sarcomas.

NDLS has demonstrated effectiveness and tolerability in the treatment of various cancers such as breast, ovarian, cervical, penile, hormone refractory prostate, non-small cell lung cancers, and sarcoma [12, 18–22]. The efficacy of NDLS in sarcoma patients (*n* = 3) was demonstrated in a previous single-center retrospective study [18]. The current multicenter retrospective study further strengthens the efficacy and tolerability data of NDLS in the treatment of sarcoma. The conventional docetaxel formulation has demonstrated effectiveness in sarcoma in previous studies [7–9]. In the benchmark study by EORTC Soft Tissue and Bone Sarcoma Group, single-agent docetaxel demonstrated an ORR of 17% in the treatment of advanced sarcoma patients (*n* = 29). Hypersensitivity, anaphylactoid reactions, fluid retention, and sensory neurotoxicity along with the hematologic AEs (neutropenia, thrombocytopenia, anemia, and leucopenia) were reported in this study [7], which are the frequently reported AEs with conventional docetaxel formulation.

NDLS was most commonly used in combination with gemcitabine in the current study. NDLS-gemcitabine combination was used in neoadjuvant setting in one patient, which demonstrated complete response. This result is similar to a previous case report, wherein neoadjuvant treatment with docetaxel and gemcitabine combination showed near-complete pathologic response in a patient with locally advanced leiomyosarcoma of the bladder [23].

In adjuvant setting, 45% patients with stages I–IV high grade uterine leiomyosarcoma were progression-free at 2 years with docetaxel-gemcitabine combination in a study (*n* = 25) by Hensley and colleagues; median OS was not reached in this study at a median follow-up of 49 months [24]. A phase III NRG Oncology/Gynecologic Oncology Group study compared adjuvant gemcitabine plus docetaxel followed by doxorubicin or observation for high-grade uterine leiomyosarcoma and showed similar treatment outcomes for both the groups [25]. In our study, 3 patients with leiomyosarcoma received NDLS/gemcitabine combination in adjuvant setting, and all the patients were still alive at the last follow-up.

In metastatic setting, the efficacy and safety of docetaxel-gemcitabine combination was evaluated in a phase III randomized GeDDiS study in patients with metastatic sarcoma (*n* = 128), which demonstrated that 46.4% patients were alive and progression-free at 24 weeks after treatment at a median follow-up of 22 months [26]. The Sarcoma Alliance for Research through Collaboration study showed an ORR of 16% and median OS of 17.9 months with docetaxel-gemcitabine treatment in patients with metastatic soft tissue sarcomas (*n* = 122) [8]. Hensley and colleagues demonstrated an ORR of 53% and median OS of 17.9 months with docetaxel and gemcitabine combination for the treatment of unresectable leiomyosarcoma (*n* = 34) [9]. In patients with metastatic or relapsed leiomyosarcoma (*n* = 24), docetaxel-gemcitabine combination resulted in an ORR of 24% in TAXOGEM study [27]. Two retrospective studies by Leu et al. (*n* = 35) and Bay et al. (*n* = 114) had ORR rates of 43% and 18%, and median OS of 13 months and 12.1 months, respectively, with docetaxel-gemcitabine combination in the treatment of sarcomas [28, 29]. The aforementioned evidence suggests an ORR of 16%–53% and a median OS of 12.1 months–17.9 months with docetaxel-gemcitabine combination in sarcoma patients in a metastatic setting.

NDLS was most commonly administered in combination with gemcitabine in metastatic setting in our study. NDLS-based chemotherapy demonstrated an ORR of 50% and a median OS of 15.8 months in metastatic setting, which is comparable with the above data. In our study, NDLS plus gemcitabine was administered to 4 patients with leiomyosarcoma in metastatic setting. The response was available in 3 patients, which showed CR, PR, and SD in one patient each; the corresponding ORR was 66.7% (2/3), comparable with that reported by Hensley et al. [9].

Overall, NDLS-based regimens were found to be well tolerated in sarcoma patients. The safety profile of NDLS in this study is consistent with previous literature [12, 13, 30]. Neutropenia, thrombocytopenia, lymphopenia, and anemia were the hematological AEs, whereas nausea, vomiting, and diarrhea were the most common nonhematological AEs. All the AEs were grade I or II. The Sarcoma Alliance for Research through Collaboration Study 002 reported that most common grade III/IV AEs with docetaxel and gemcitabine combination in patients with metastatic soft tissue sarcoma (*n* = 122) were thrombocytopenia (40%), fatigue (16%), and febrile neutropenia (5%) [8]. The SARC phase III study showed a significantly higher rate of discontinuation due to toxicity in patients receiving docetaxel and gemcitabine combination versus gemcitabine alone (*P* < 0.01) [31]. In the current report, grade III/IV AEs were not reported with NDLS and gemcitabine combination. The major limitation of this study due to its retrospective nature is data availability with respect to survival and safety and the small pool of patients.

Overall, NDLS-based chemotherapy was effective and well tolerated in managing sarcoma. These real-world data provide valuable insights into the effectiveness and safety of NDLS as a potential treatment option in the management of sarcoma, while these results need to be established in a larger population in prospective trials.

## Figures and Tables

**Table 1:** Patient disposition and baseline characteristics.

Parameters	All patients (*N* = 11)	Neoadjuvant setting (*N* = 1)	Adjuvant setting (*N* = 3)	Metastatic setting (*N* = 7)
Age (years), mean ± SD, range	46.09 ± 11.46 (19–59)	48	44 ± 10.15 (35–55)	46.71 ± 13.46 (19–59)
BSA, kg/m^2^, mean ± SD	1.63 ± 0.20	1.62	1.57 ± 0.18	1.66 ± 0.23
Gender, *n* (%)				
Men	4 (36.4)	1 (100)	2 (66.7)	1 (14.3)
Women	7 (63.6)	—	1 (33.3)	6 (85.7)
Cancer stage, *n* (%)				
II	3 (27.3)	—	3 (100)	—
III	1 (9.1)	1 (100)	—	—
IV	7 (63.6)	—	—	7 (100)
Metastasis site, *n* (%)				
Lungs	—	—	—	3 (42.9)
Lymph node	—	—	—	2 (18.2)
Bone	—	—	—	1 (14.3)
Brain	—	—	—	1 (14.3)
ECOG performance score				
1	4 (36.3)	—	1 (33.3)	3 (42.9)
2	7 (63.7)	1 (100)	2 (66.7)	4 (57.1)
Comorbid disease, *n* (%)				
Hypertension	5 (45.5)	1 (100)	1 (33.3)	3 (42.9)
Diabetes	1 (9.1)	—	—	1 (14.3)
Hypothyroidism	1 (9.1)	—	—	1 (14.3)

Abbreviations: BSA, body surface area; ECOG, Eastern Cooperative Oncology Group; SD, standard deviation.

**Table 2:** Patient details with chemotherapy regimens and efficacy evaluation.

No.	Age	Sex	If others, please specify	Cancer stage	Setting in which NDLS was used	NDLS chemotherapy used	Overall response as per the RECIST (1.1)
1	48	Male	High-grade pleomorphic sarcoma of mandible	III	Neoadjuvant setting	NDLS plus gemcitabine	CR
2	42	Male	Leiomyosarcoma	II	Adjuvant setting	NDLS plus gemcitabine	Not applicable
3	35	Male	Leiomyosarcoma	II	Adjuvant setting	NDLS plus gemcitabine	Not applicable
4	55	Female	Leiomyosarcoma	II	Adjuvant setting	NDLS plus gemcitabine	Not applicable
5	49	Female	Leiomyosarcoma	IV	Metastatic setting	NDLS plus gemcitabine	NE
6	44	Female	Leiomyosarcoma	IV	Metastatic setting	NDLS plus gemcitabine	SD
7	46	Female	Leiomyosarcoma	IV	Metastatic setting	NDLS plus gemcitabine	CR
8	58	Female	Leiomyosarcoma	IV	Metastatic setting	NDLS plus gemcitabine	PR
9	59	Male	Malignant fibrous histiocytoma of right thigh	IV	Metastatic setting	NDLS plus gemcitabine	PR
10	52	Female	Extraskeletal myxoid chondrosarcoma	IV	Metastatic setting	NDLS plus cyclophosphamide	PD
11	19	Female	Osteosarcoma of left femur	IV	Metastatic setting	NDLS plus gemcitabine	PD

*Note*. Efficacy response was not evaluated for patients in adjuvant setting. Abbreviations: CR, complete response; PR, partial response; SD, stable disease; PD, progressive disease; NDLS, nanosomal docetaxel lipid suspension; NE, not evaluated.

**Table 3:** Safety profile of NDLS based chemotherapy in sarcoma (*n* = 11).

Adverse event	All grades, *n* (%)	Grade I, *n* (%)	Grade II, *n* (%)
Hematological AEs			
Neutropenia	2 (18.2)		2 (18.2)
Thrombocytopenia	1 (9.1)	1 (9.1)	
Anemia	1 (9.1)	1 (9.1)	
Lymphopenia	1 (9.1)	1 (9.1)	
Nonhematological AEs			
Nausea	4 (36.4)	4 (36.4)	
Vomiting	4 (36.4)	4 (36.4)	
Diarrhea	3 (27.3)	3 (27.3)	
Mucositis	2 (18.2)		2 (18.2)
Mouth ulcer	2 (18.2)	2 (18.2)	
Weakness	1 (9.1)		
Alopecia	1 (9.1)	1 (9.1)	

*Note*. AEs in different grades may occur in ≥1 patient; hence, the number of cumulative number of patients in different grades may exceed the total number of patients with individual AEs.

## Data Availability

The datasets analyzed to support the findings of this study are available from the corresponding author upon request.
